# The Role of RhoH in TCR Signalling and Its Involvement in Diseases

**DOI:** 10.3390/cells10040950

**Published:** 2021-04-20

**Authors:** Ana Masara Ahmad Mokhtar, Ilie Fadzilah Hashim, Muaz Mohd Zaini Makhtar, Nor Hawani Salikin, Syafinaz Amin-Nordin

**Affiliations:** 1 Bioprocess Technology Division, School of Industrial Technology, Universiti Sains Malaysia, Gelugor 11800, Penang, Malaysia; muazzaini@usm.my (M.M.Z.M.); norhawani@usm.my (N.H.S.); 2 Primary Immunodeficiency Diseases Group, Regenerative Medicine Cluster, Advanced Medical and Dental Institute, Universiti Sains Malaysia, Kepala Batas 13200, Penang, Malaysia; iliehashim@usm.my; 3 Department of Medical Microbiology, Faculty of Medicine and Health Sciences, Universiti Putra Malaysia, Serdang 43400, Selangor, Malaysia; syafinaz@upm.edu.my

**Keywords:** small GTPase, RhoH, malignant B cell, immunodeficiency, autoimmune

## Abstract

As an atypical member of the Rho family small GTPases, RhoH shares less than 50% sequence similarity with other members, and its expression is commonly observed in the haematopoietic lineage. To date, RhoH function was observed in regulating T cell receptor signalling, and less is known in other haematopoietic cells. Its activation may not rely on the standard GDP/GTP cycling of small G proteins and is thought to be constitutively active because critical amino acids involved in GTP hydrolysis are absent. Alternatively, its activation can be regulated by other types of regulation, including lysosomal degradation, somatic mutation and transcriptional repressor, which also results in an altered protein expression. Aberrant protein expression of RhoH has been implicated not only in B cell malignancies but also in immune-related diseases, such as primary immunodeficiencies, systemic lupus erythematosus and psoriasis, wherein its involvement may provide the link between immune-related diseases and cancer. RhoH association with these diseases involves several other players, including its interacting partner, ZAP−70; activation regulators, Vav1 and RhoGDI and other small GTPases, such as RhoA, Rac1 and Cdc42. As such, RhoH and its associated proteins are potential attack points, especially in the treatment of cancer and immune-related diseases.

## 1. Introduction

The Rho (Ras homologous) family of small GTPases is one of the members of the Ras superfamily. In humans, 20 Rho-family GTPase members have been identified and categorised into eight major subfamilies Rho-, Rac-, Cdc42-, RhoU/RhoV-(also known as Wrch/Wrch2), Rnd-, RhoD/RhoF-, RhoBTB- and RhoH subfamilies. The subfamilies are categorised based on the similarities in their primary amino acid sequence (Table 1), structural motifs and biological functions [1]. Initially, Miro and RhoBTB3 were included as members of the Rho family small GTPases, but they have been excluded due to the lack of similarity between these proteins and other members of the Rho-family. For instance, Miro subfamily proteins lack the Rho insert region and are assumed to be non-catalytic as the sequence in the highly conserved G2–G5 loops responsible for nucleotide binding and hydrolysis is different compared with that in canonical GTPase domains [2,3]. RhoBTB3 shares 25% sequence similarity with and contains the same BTB domains as RhoBTB1 and RhoBTB2. However, this protein is not considered as a Rho GTPase by some studies due to its lack of similarity with Rho and Ras [3,4].

Compared with other Ras superfamily proteins, the Rho-family small G proteins have a Rho insert region, which is a unique α-helical sequence located between the fifth β strand and the fourth α helix in the G domain [5]. Many of the typical Rho-family GTPases only have a Rho-type G domain with short N- and C-terminal extensions [6,7]. Similar to other Ras superfamily members, they also have a hypervariable region encoding a prenylation site at their C-termini and sometimes a polybasic region. These signals allow them to associate with specific membrane compartments [8].

Additionally, some of them also possess a CAAX (C: Cys, A: aliphatic residue, X: any amino acid) tetrapeptide sequence at their C-termini, which is important for lipid modification. This process involves three main steps, which are isoprenylation, proteolysis and carboxyl methylation [9]. An isoprenoid lipid is attached to the CAAX box by a prenyltransferase, such as geranylgeranyltransferase (GGTase) or farnesyltransferase (FTase). Both of these enzymes recognise the CAAX sequence before adding a 20-carbon geranylgeranyl or 15-carbon farnesyl to the cysteine residue of the CAAX sequence via a thioether linkage. Prenylation is followed by the proteolysis of the three C-terminal residues (AAX) by a prenyl protein peptidase (from the Rce1 family as an example) to release AAX. The prenylated cysteine is then methylated by isoprenyl-cysteine carboxyl methyl-transferase. Compared with the atypical small RhoGTPases, many of the typical Rho-family GTPases are geranyl-geranylated or farnesylated, and only a few, such as RhoB, TC10 and Rac1, are palmitoylated (Table 2) [8,10]. However, some of the atypical small Rho GTPases also have a functional CAAX box that allows them to undergo prenylation (Table 2). For instance, both RhoU and RhoV only undergo palmitoylation, while Rnd proteins undergo farnesylation. Conversely, the RhoBTB subfamily does not possess any canonical CAAX motifs, suggesting that they may not undergo any lipid modification at the C-terminal.

Among the Rho-family GTPases, the most extensively studied members are RhoA, Rac1 and Cdc42. These proteins and their subfamilies are known as classical or typical Rho-family GTPases. The other Rho-family proteins are known as the non-classical or atypical Rho GTPases and include the RhoBTB and Rnd subfamilies. This classification is made based on the ability of the Rho-family GTPases to undergo the standard GTPase cycle [6,13]. The atypical Rho GTPases often contain extra domains and are therefore longer than classical Rho GTPases.

Compared with the atypical small GTPases are further divided into fast-cycling and GTPase-defective G proteins (Table 3). In resting cells, the typical Rho-family GTPases are generally assumed to be in their inactive GDP-bound form either alone or in complex with RhoGDIs [14]. However, in contrast to the typical Rho-family GTPases, RhoD, RhoF, RhoU and RhoV have a rapid nucleotide exchange compared with standard GTP hydrolysis, which classifies them as fast-cycling small G proteins [15,16,17]. The Phe28 mutations of Ras, Rac1, Cdc42 and RhoA have rapid nucleotide exchange and can induce oncogenic transformation [18,19]. RhoU and RhoV also have mutated phenylalanine at position 28 (Table 3), and this factor might be one of the reasons for considering both of these proteins as fast-cycling small G proteins.

The fast-cycling Rho GTPases have GTPase activity and are identical to Cdc42 at amino acids Gly12, Ala59 and Gln61 (Ras numbering) (Table 3) [13]. Gly12 and Ala59 are important for GTP hydrolysis, while Gln61 is necessary to stabilise the transition state of hydrolysis [20]. These amino acid residues are reported to be mutated in GTPase-deficient Ras [21]. By contrast, the GTPase defective atypical Rho-family members, including Rnd1, Rnd2, Rnd3, RhoBTB1, RhoBTB2 and RhoH, do not possess the conserved Gly12, Ala59 and Gln61 compared with Cdc42 (Table 3) [4,13].

Most studies have focused on the role of the typical small Rho GTPases in diseases, but information on the atypical small Rho GTPases is limited. Among the rare small Rho GTPases that receive much attention is RhoH as it is associated with several diseases, such as B cell malignancies, immunodeficiency and autoimmune diseases. This small GTPase has a unique role compared with others because it acts as a tumour-suppressor protein. Hence, a thorough understanding of the function of RhoH in both normal and disease conditions may help to identify and design an efficacious target.

## 2. RhoH, An Atypical Rho Family Small GTPase

**Figure 1:**
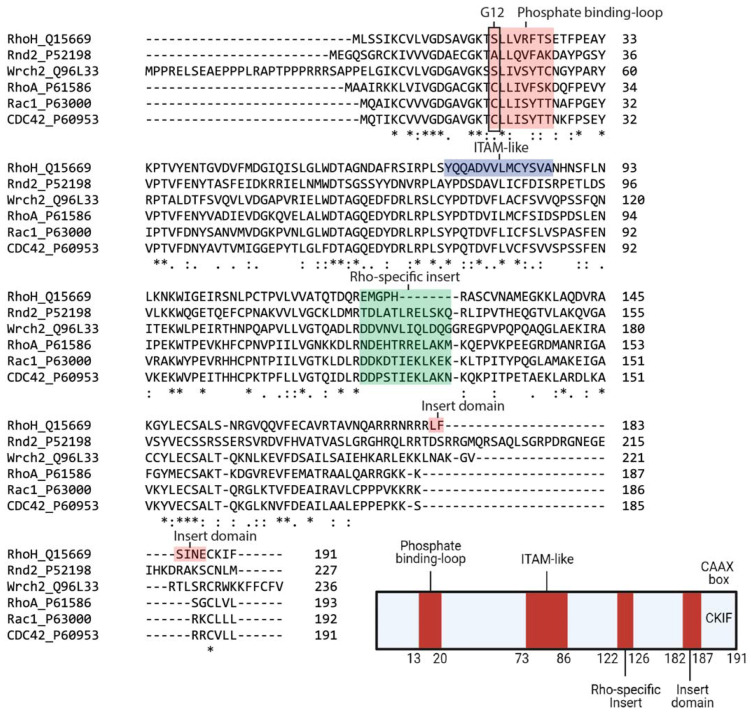
Sequence alignment and architecture of RhoH. Sequence alignment of RhoH, Rnd2, Wrch2, RhoA, Rac1 and Cdc42. Rnd2 and Wrch2 are representatives of the fast-cycling and GTPase-defective small GTPases, while RhoA, Rac1 and Cdc42 are typical small Rho GTPases (**Top**). RhoH shares a phosphate-binding loop with other small Rho GTPases but is assumed as GTPase defective due to the absence of Gly12. The Rho-specific insert in RhoH is shorter than that in other small Rho GTPases. RhoH also has an ITAM-like motif at position Tyr73 to Ala86 and a carboxyl-terminal insert domain (L_182_FSINE_187_) that function to regulate RhoH stability. RhoH also undergoes prenylation at the C-terminal due to the presence of the CAAX box (CKIF), similar to other typical small Rho GTPases (**Bottom**). (Created with Biorender).

Within the family, RhoH and Cdc42 share 42% sequence similarity, with the major differences in the C-terminal region (Figure 1). It also contains a shorter Rho insert region that is more similar to classical Rho-family proteins but is GTPase defective because conserved residues corresponding to Gly12, Ala59 and Gln61 (Ras numbering) are absent (Table 3). However, RhoH has a CAAX box, a CKIF motif at its C-terminus, which allows farnesylation and geranyl-geranylation [22] and facilitates membrane targeting. RhoH myristoylation at the N-terminal may also assist RhoH attachment to the plasma membrane and promote its interacting partners, Lck, ZAP−70 and Syk recruitment to the membrane to facilitate T cell receptor (TCR) signalling [23,24,25]. Among the Rho family members, RhoH is the only member with an ITAM-like motif, which is characterised by two tyrosines, spaced by six amino acids: Y73xxA76_6_Y83xxA86 (Figure 1). This consensus motif is crucial for the recruitment of ZAP−70 to CD3ζ in the immunological synapse [23].

Given that RhoH may be GTPase defective, other mechanisms have been described to regulate RhoH activity, including the regulation of mRNA level and tyrosine phosphorylation of the unique ITAM-like motif [22]. Additionally, RhoH activity can be regulated by lysosomal degradation through its unique C-terminal region, L_182_FSINE_187_ domain, located in between its polybasic region and CAAX box. This insert domain has been shown to regulate RhoH stability via chaperone-mediated autophagy (CMA) [26].

In contrast to the typical Rho-family members, which are best studied for their role in promoting actin cytoskeleton reorganisation, especially during cell division and migration [2], RhoH does not regulate actin reorganisation in NIH3T3 or MDCK cells [27]. However, Mino et al., (2018) showed that RhoH is involved in modulating the structure of actin-cytoskeleton and transcriptional activity during T cell migration and adhesion by forming a multi-protein complex with p120 catenin and the transcriptional regulator, Kaiso, to attenuate Rac1 signalling. Aside from regulating Rac1 signalling, RhoH interaction with Kaiso is necessary to facilitate Kaiso nuclear localisation to repress *BCL6* gene, a transcriptional repressor expression, resulting in an increase in tumour suppressor p53 protein [28]. Collectively, the inhibitory function of RhoH would help to reduce the events of cell survival, migration and invasion [29,30]. By contrast, although RhoH is assumed to be expressed only in haematopoietic cells, it was shown to promote cell migratory polarity in prostate cancer cell line by directing Rac1 and PAK2 to membrane protrusions [31]. These contrary functions of RhoH might be dependent on the type and origin of the cells. For instance, RhoH is dispensable for the development of myeloid, erythroid, and B cells but is crucial for T cell production, survival and migration [32,33]. This function is consistent with the study of Chae et al., (2010) that showed a lack of RhoH-impaired thymocyte development and stimulation of peripheral T cell unresponsiveness [23,34].

**Figure 2:**
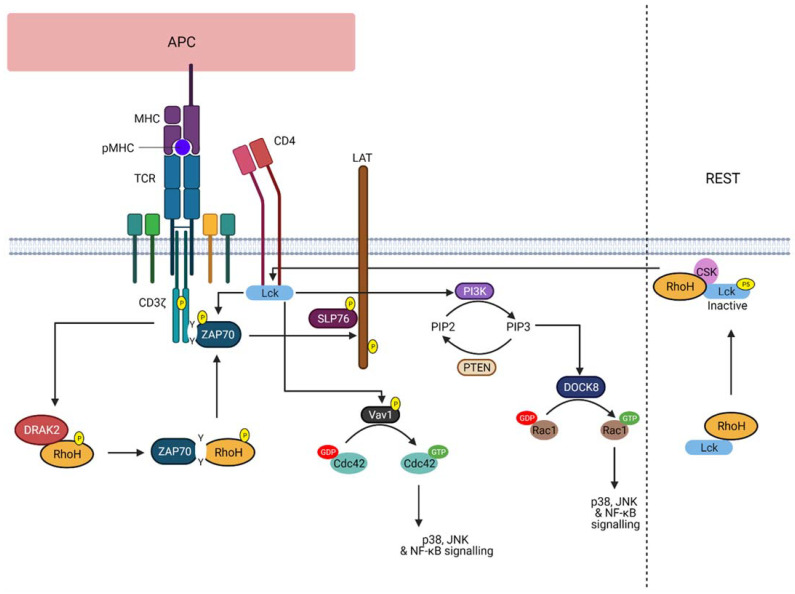
RhoH function upon TCR activation. The illustration shows the role of RhoH in regulating TCR signalling. Upon TCR stimulation, RhoH is phosphorylated by DRAK2 that promotes the interaction between RhoH and ZAP−70 via the ITAM-like motif of RhoH. ZAP−70 is recruited to the TCR CD3ζ chain, thereby activating LAT and SLP76 signalling. As for Lck, RhoH functions to assist Lck recruitment to the membrane to facilitate TCR signalling. Active Lck promotes PI3K signalling and PIP3-induced DOCK8 activation. As a result, Rac1-related signalling is activated. Vav1-induced Cdc42 activation can also be regulated by Lck. However, at rest, RhoH recruits Lck to the plasma membrane, where RhoH accelerates and supports the inhibitory phosphorylation of Lck at Y505 (labelled with P5) by CSK (Created with Biorender).

The current knowledge of RhoH function is only limited in T cells (Figure 2), whereupon during response to TCR activation, RhoH is phosphorylated by kinases, such as DRAK2 [35]. This phosphorylation event promotes its interaction with ZAP−70 via the ITAM-like motif. ZAP−70 and Lck will then be recruited to the TCR, where ZAP−70 is activated and colocalised with its substrates. As a result, activated ZAP−70 will promote linker of activated T cells (LAT) and SLP76 signalling in T cell, crucial for T cell activation and development [23,24,25,36]. Additionally, RhoH was also found to act as an adaptor protein that retains Lck in an inactive state, thereby suggesting that RhoH can regulate both pre-TCR and TCR signalling during T cell development. However, in response to ligand-mediated TCR activation, Lck is recruited to the membrane by RhoH and dephosphorylated by CD45, resulting in Lck auto-activation and its release from RhoH [37]. Active Lck will then stimulate PI3K signalling and the activity of RhoGEF, Vav1. Nonetheless, despite the obvious role of RhoH in T cell development, RhoH involvement in T cell-specific malignancies, such as in T cell lymphomas, is not well understood. Conversely, RhoH is known to be associated with B-cell neoplasm, but its function in regulating B cell signalling should be further studied.

## 3. Deregulation of RhoH in Diseases

### 3.1. RhoH in B-Cell Malignancies

*RhoH*/TTF gene was initially discovered as a translocation partner of *LAZ3/BCL6* gene in a non-Hodgkin’s lymphoma cell line, VAL t (3;4) (q27;p23), and within the *IgH* gene, t (4;14) (p13;p32), in patients with multiple melanoma [38]. These chromosomal alterations of *RhoH* gene result in the aberrant expression of RhoH, and disruption of *RHOH* has been reported in various lymphoma cases [39,40]. However, 46% of cases are observed in diffuse large B cell lymphoma (DLBCL) and are found to be related to somatic hypermutations in the noncoding region, rather than chromosomal rearrangements [41]. SHM occurs during B cell development to generate high-affinity antibodies and may also aberrantly target several proto-oncogene genes, including RhoH, wherein this loss-of-function mutation was observed in 50% cases of diffuse large-cell lymphomas (DLCLs) [42]. However, RhoH aberrant somatic hypermutation (aSHM) rarely occurs in Burkitt lymphoma and follicular lymphoma (FL), suggesting that RhoH aSHM selectively occurs depending on the type of lymphoma and most probably in more aggressive types. RhoH aSHM was found to contribute to cancer progression from FL or chronic lymphocytic leukaemia (CLL) to DLBCL, indicating that deregulation of RhoH protein expression is highly related to higher-grade lymphomas and might act as an important prognostic marker [43,44]. In DLBCL, loss of RhoH was associated with impaired kaiso nuclear localisation and increased BCL6 protein expression [28]. Consistent with these findings, constitutively expressed BCL6 was found to stimulate B cell proliferation and germinal centre (GC) formation, which are characteristics of the typical human DLBCL [45].

The involvement of RhoH aSHM mutation in lymphoma progression is highly correlated with the mRNA levels of activation-induced deaminase (AID) enzyme [43], wherein low levels of AID mRNA expression result in the low frequency of RhoH aSHM. Similarly, in the murine model of acute lymphoblastic leukaemia (ALL), loss of AID shows a low frequency of RhoH aSHM [46]. Additionally, high mRNA levels of RhoH were observed, indicating that RhoH expression is tightly regulated by AID-induced aSHM. Interestingly, RhoH is widely known as a tumour suppressor, suggesting that deregulation of RhoH activity may influence cancer progression [22]. While other small Rho GTPases promote cell division and inflammatory response via NF-κB signalling [47,48,49], RhoH negatively regulates cancer-related signalling by inhibiting IκB degradation [22]. RhoH can also regulate Rac1 subcellular localisation at the cell periphery and inhibit the Rac1-induced activation of p38 MAPK and NF-κB in Jurkat cells, causing deregulation in cell death and metastasis [27,31]. RhoH inhibits Rac1-induced Bad, a pro-apoptotic protein phosphorylation at Ser78, leading to the inhibition of apoptosis event [50]. Downregulation of Rac1 activity by RhoH might be due to its role in enhancing the inactivation phosphorylation of Lck by C-terminal Src kinase (CSK) [37]. Inactive Lck will then fail to stimulate PI3K signalling [51] and decrease DOCK8-induced Rac1 activation [52]. Additionally, Lck regulates another RhoGEF known as Vav1, wherein this regulation may influence RhoA activation [53]. RhoH-induced GATA−2 expression during eosinophil differentiation also requires ROCK, an effector protein for RhoA, suggesting that both RhoH and RhoA crosstalk [54,55,56]. However, no direct association has been established between RhoH and ROCK, but because RhoH is known to negatively regulate Rac1 activity [27], RhoH may alleviate the Rac1-mediated antagonism of RhoA/ROCK signalling [57]. Thus, low levels of RhoH resulting from aSHM might directly or indirectly promote Rac1-, RhoA- or Cdc42-induced cell division and migration, leading to an increase in cancer progression.

Aside from aSHM, low levels of RhoH were also observed in several patients with hairy cell leukaemia (HCL), acute myeloid leukaemia (AML) and CLL [50,58,59] but the mechanism involved in this protein levels deregulation is still unknown. However, because RhoH expression can be regulated by phosphorylation, lysosomal degradation and transcriptional repression by the AP−1 transcription factor, JunD [22,60,61], either of these regulations may be impaired in leukaemia. For instance, JunD protein levels are high in adult T cell leukaemia (ATL) and are related to the increase in cell proliferation and anchorage-independent growth [62]. Consistent with this finding, overexpression of JunD was found to repress *RhoH* gene in AML [61,63], indicating that regulation of *RhoH* mRNA expression by a transcriptional repressor may affect RhoH anti-tumour activity.

Information on the role of RhoH in the pathogenesis of leukaemia is limited, but it is known to reduce HCL proliferation and transendothelial migration in a xenograft mouse model of HCL [58]. Loss of RhoH protein expression was found to promote CD11c surface expression, contributing to CLL and HCL aetiopathogenesis [64,65]. In the case of AML, lack of RhoH is presumed to contribute to AML pathogenesis by inhibiting terminal myeloid differentiation [63]. Interestingly, RhoH-induced terminal myeloid differentiation may require Cdc42 and/or Wnt signalling inhibition because 13 affected genes were identified following *RHOH* induction. The downregulated proteins are Borg2, Borg5, PKCζ, FOXK1, SRC, PKCε, BMAL1, TBL1X and TBL1Y, which are known to stimulate Cdc42 and/or Wnt signalling. Meanwhile, the four other proteins that act as negative regulators or antagonists for signalling are NKD2, Sep11, FAT1 and PICK1. Interestingly, all these proteins are critically involved in the pathogenesis of AML as they influence the functional characteristics of mature myeloid cells [63]. Additionally, RhoH induction was also found to decrease CD11b and CD93 protein expression, which is observed in terminally differentiated macrophage [63,66,67]. Collectively, RhoH activity in the myeloid cell is critical in promoting macrophage terminal differentiation by the repression of the interlinked Cdc42 and Wnt intracellular signalling pathways and inflammatory effector proteins, CD11b and CD93. As such, RhoH may be used in alternate AML differentiation therapy because it can stimulate myeloblast differentiation that is often blocked in AML [63].

Despite several studies that correlate the low protein levels of RhoH with cancer progression, increased RhoH protein expression was also found to contribute to malignancy. For instance, RhoH protein expression was augmented in a transgenic mouse model of CLL and positively correlated with disease progression [33]. This finding is attributed to an increased RhoH-induced ZAP−70 activity, especially on TLR9-induced IgM secretion and degradation of pro-apoptotic protein, BIM, both of which are features of BCR signalling [33,68]. Constitutively active B cell receptor (BCR) signalling will then promote AKT-induced cell survival and a more aggressive lymphoma [69,70].

Overall, deregulation of RhoH protein expression can modulate the activity of several proteins, including the other small Rho GTPases, which are known to be significantly involved in cell survival and proliferation, thus promoting cancer progression. As such, the role of RhoH as a modulator of signalling cascades should be further elucidated to understand its biological implications in patients with cancer.

### 3.2. Immune-Related Diseases

#### 3.2.1. Primary Immunodeficiencies (PIDs)

PIDs are clinically and genetically heterogeneous disorders that involve the mutations of more than 400 genes [71]. Patients with PIDs usually present with severe and/or unusual infections and autoimmune and lymphoproliferative. Among the 400 mutated genes identified in PIDs, RhoH has been found in two patients with PID, particularly in patients with epidermodysplasia verruciformis (EV). Both of the patients have RhoH mutation at Y38X, which results in the generation of a stop codon in RhoH and loss-of-function effect. Loss of RhoH function contributes to the lack of naïve peripheral T cell and persistent EV-HPV infections, as presented by the patients [72]. A defective T cell development and function are predicted because RhoH is required for thymocyte selection and maturation by mediating TCR-induced ZAP−70 activation and CD3ζ phosphorylation [32,34]. This defect arose due to the failure of RhoH in recruiting ZAP−70 and Lck to CD3ζ in the immunological synapse [23], indicating its role in pre-TCR and TCR signalling [37].

Interestingly, one of the RhoH-deficient patients also developed Burkitt lymphoma, suggesting the role of RhoH in regulating cancer development in an immunocompetent patient. The same lymphoproliferative events were also observed in several immunodeficiency-associated disorders, such as in the B cell post-transplant lymphoproliferative disorder [73] and AIDS-associated non-Hodgkin lymphoma [74], wherein both cases of immunocompetent patients were reported to have low levels of RhoH due to aSHM. The immune system may be involved in identifying and eliminating nascent cancer [75,76]. This assumption is based on the specific antigens of cancer cells that can be recognised by the immune system. However, patients who have weakened or deficient immune systems cannot eliminate cancer cells, resulting in a state of equilibrium. In this state, cancer cells do not progress and further metastasise but eventually, they may be able to avoid and resist the anti-tumour immune response, leading to tumour progression [75,77]. Thus, these immunocompetent patients have a higher tendency than healthy individuals to develop cancers.

**Figure 3:**
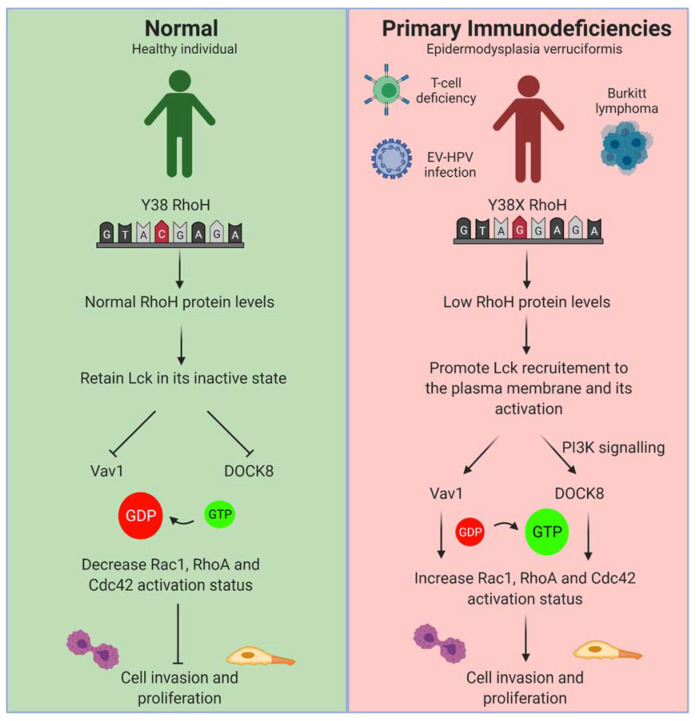
Potential role of RhoH in PID malignancy. RhoH Y38X mutation has been found in PIDs particularly in patients with epidermodysplasia verruciformis (EV). The patient was shown to have T cell deficiency, increased susceptibility to human papillomavirus (HPV), and Burkitt lymphoma. The exact mechanism for cancer development in patients with PID is still unknown but might be due to the ability of RhoH to regulate other cancer-related small Rho GTPase activities, including RhoA, Cdc42 and Rac1. This deregulation can be achieved through Lck, wherein loss of RhoH may promote Lck recruitment to the plasma membrane and promote its activation in response to TCR stimulation (Created with Biorender).

The exact mechanism for this cancer development is still not well understood, but it might be due to the ability of RhoH to regulate the activities of other cancer-related small Rho GTPases, including RhoA, Cdc42, Rac1 and RhoF [47,78,79,80] (Figure 3). This prediction is consistent with the data provided by Itan and Casanova, (2015) illustrating that several novel Rho small GTPase genes, including RhoA, RhoB, Rac1, RhoF, RhoD, RhoG, RhoV and RhoBTB, are associated with RhoH in PIDs [81]. This crosstalk might involve the regulatory proteins, RhoGEF and RhoGDI. For instance, RhoH Y38X mutation may promote Lck-induced PI3K signalling and Rac1-specific GEF, DOCK8 activity toward Rac1 [37,82]. Downregulation of RhoH may also promote Lck-induced Vav1 activity, increasing Cdc42 activation [83]. However, Dorn et al. (2007) found a positive correlation between RhoH and Vav1, wherein increased protein expression of RhoH promoted Vav1 GEF activity toward its target, Rac1, RhoA and Cdc42 [32]. This result might be due to RhoH that does not only retain Lck in the inactive state but also functions to recruit Lck to the plasma membrane following TCR stimulation. Additionally, the observed lymphoproliferative event might be mediated by RhoGDIs, such as RhoGDI−3, a negative regulator of small Rho GTPases. This event is due to its role not only as an inhibitory regulator but also as a chaperone, targeting them to the specific cellular compartment [84]. RhoH and most of the predicted small Rho GTPases, including Rac1, RhoA, RhoF, RhoD, RhoG, RhoB and Cdc42, were also shown to interact with RhoGDI−3 [27,85]; this role suggests that the same interacting protein shared by these small Rho GTPases might underpin RhoH Y38X-induced lymphoproliferation. However, these assumptions require further validation.

#### 3.2.2. Autoimmune-Related Diseases

Besides involved in PIDs, lack of RhoH was also shown to induce psoriasis-like chromatic dermatitis by stimulating T cell differentiation into T_H_17 cells [86]. Psoriasis is a chronic immune-mediated inflammatory skin disease characterised by the formation of hard scaly plaques and increased keratinocyte proliferation [87,88]. The role of RhoH in psoriasis might be due to its ability to regulate T cell development [89] as deregulation of T cell causes aberrant production of psoriatic cytokines IL−17, IFN-γ, TNF, and IL−22 [87]. Although the number of peripheral mature T cell decreases in RhoH-null mice [34], the number of T_H_17 cells was shown to increase in the RhoH^−/−^ mice with dermatitis symptoms, causing an increase in IL−22, IL−23 and IL−17 production. This phenomenon is different in T cell development observed by the lack of RhoH that might be contributed by the deregulation of RORγt-induced cytokine production [86]. Similarly, Oda et al., (2013) found the development of unconventional T cell subsets, especially TCRαβ CD8αα, is not critically dependent on RhoH-mediated TCR signalling but is cytokine dependent. Therefore, modulating RhoH-T_H_17 may also serve as a therapeutic strategy for the treatment of psoriasis [89].

**Figure 4:**
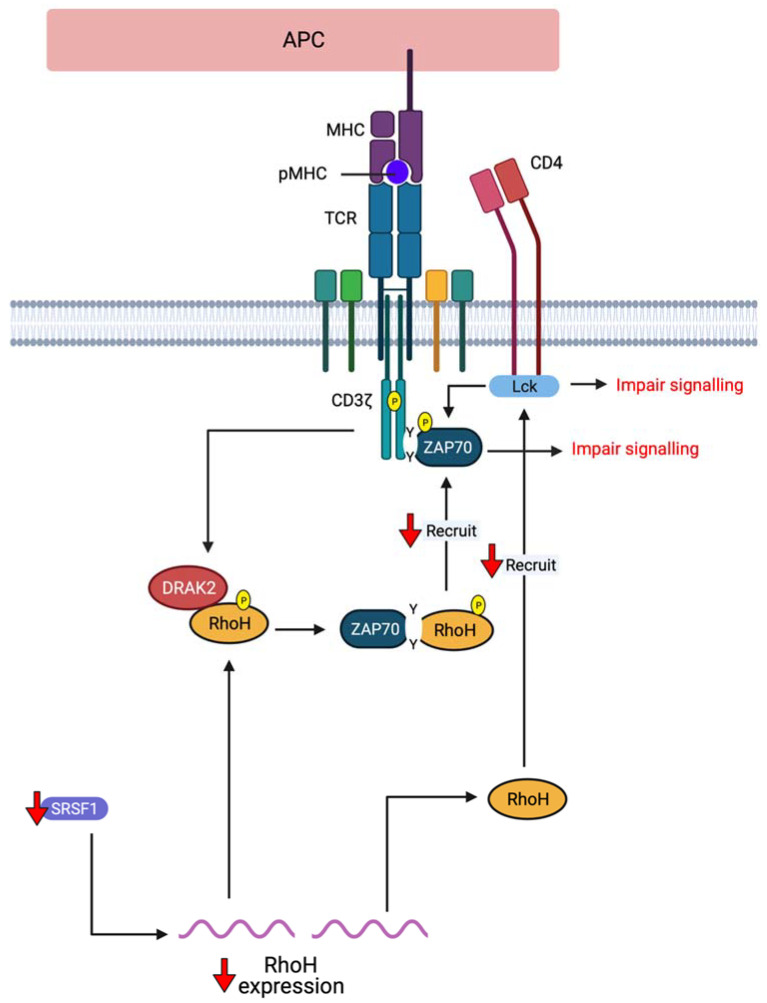
Involvement of RhoH in SLE: Low protein levels of SRSF1 were observed in the T cells of patients with SLE and is positively correlated with RhoH protein levels. Lack of RhoH will decrease the recruitment of ZAP−70 and Lck to lipid raft, causing an impaired TCR signalling (Created with Biorender).

Another potential immune-related is systemic lupus erythematosus (SLE). SLE is one of the most common systemic autoimmune diseases characterised by highly variable clinical presentations that may range from mild skin involvement to life-threatening multi-organ failure [90]. It represents a group of disorders that afflicts a specific target organ or multiple organs and can cause a burden to the use of medical care and affect the patient’s quality of life [91]. A recent study by Katsuyama et al., (2021) found the involvement of RhoH in SLE and is mediated by the serine/arginine-rich splicing factor 1 (SRSF1) (Figure 4) [92]. Low protein levels of SRSF1 were observed in the T cells of patients with SLE, causing an increase in Th1-induced IFN-γ production. An increment in IFN-γ will then enhance the inflammatory responses and contribute to the pathogenesis of SLE and lupus nephritis [93]. Surprisingly, RhoH protein levels were also shown to decrease in SRSF1-deficient T cells, and these low levels are correlated with increased production of IFN-γ, as supported by the findings of Tamehiro et al., (2019) [86]. As such, the involvement of RhoH in autoimmune-related diseases might be due to its role in regulating T helper cell-induced cytokine production.

Interestingly, IFN-γ overproduction is involved in cancer progression by stimulating tumour growth with immunoevasive properties [94]. This pro-oncogenic property of IFN-γ is probably due to its ability to regulate B cell-activating factor, BAFF production [95,96], a cytokine involved in B cell survival and differentiation. Increased production of BAFF was observed in SLE, particularly in the pathogenesis of lupus nephritis, causing an overproduction of T cell-dependent B cell pathogenic autoantibodies [95,97]. Increased BAFF protein levels were found in several malignant B cells, such as non-Hodgkin lymphoma and follicular lymphoma, wherein this elevated cell survival is promoted through the NF-κB signalling pathway [98,99]. Thus, the involvement of RhoH-induced IFN-γ production and subsequently BAFF production may underpin lupus malignancy that probably occurs through the modulation of NF-κB activation.

Meanwhile, several RhoH interacting proteins have been implicated in SLE aetiopathogenesis (Figure 4). For example, aberrant recruitment of Lyn, Syk, Lck and ZAP−70 into the lipid raft of activated B and T cells were shown to contribute to SLE aetiopathogenesis [100,101]. In healthy individuals, ZAP−70, Syk and Lck recruitment to lipid raft is increased, whereas, in patients with SLE, their recruitment is impaired. This deregulation might be modulated by RhoH as it is known to be critically involved in the recruitment of ZAP−70 and Lck to CD3ζ and the immunological synapse of T cell and Syk recruitment to the membrane for signal transduction [23,24,25]. As such, TCR activation would be impaired that might reduce peripheral tolerance [102]. Although RhoH function is only characterised in T cells, RhoH may act the same in B cells. This phenomenon is due to the presence of ZAP−70, Syk and Lck in both T and B cell lipid raft, and this raft is implicated in the organisation of T cell immunological synapse [103,104].

Additionally, increased Notch1 signalling was observed in the SLE murine model [105] and is potentially due to the increase in Notch1 protein expression by BCR ligation [106]. Increased Notch1 signalling will then contribute to B cell activation, development and differentiation and are common characteristics of SLE [107,108]. Interestingly, ZAP−70 may participate in BCR signalling by limiting the ability of BCR to induce negative B-cell selection and cell death [109]. Additionally, ZAP−70 may regulate BCR signalling via its kinase activity or as a scaffold protein recruiting the other tyrosine kinases [110]. Given that RhoH is widely known to regulate ZAP−70 activation, it may also be implicated in SLE. However, these assumptions require further analysis.

Taken together, RhoH and its interacting partners may serve as valuable targets for the effective treatment of autoimmune-related diseases, including PIDs, psoriasis and SLE.

## 4. RhoH as a Therapeutic Target

Considering the importance of RhoH in regulating TCR and BCR signalling pathways, much effort has been exerted on targeting RhoH to control diseases derived from abnormal T and B cell activation, such as cancer, immunodeficiency disorders and autoimmune diseases. RhoH protein levels are frequently reduced in the aforementioned diseases, suggesting the need to rectify the aberrant RhoH expression in the haematopoietic cells. The current drugs known to modulate RhoH activities are lenalidomide and ibrutinib [111]. However, the use of these drugs is critically dependent on the types of disease as these drug treatments were shown to regulate RhoH protein expression. For instance, the use of lenalidomide in the CLL murine model results in a decreased RhoH protein expression that promotes Rac1 and RhoA activation [59]. Additionally, deregulated RhoH protein expression can be corrected by using the current approach that relies on the potential therapeutic opportunity to target CMA [112]. This factor is based on the understanding that cellular protein levels are significantly influenced by protein stability. Hence, regulating their modes of degradation may promote protein stability. For instance, all-*trans* retinoic acid can be used to block the inhibitory effect of retinoic acid receptor alpha (RARα) on the CMA process, especially on LAMP2A expression and its trafficking to lysosomes [113]. This approach can be applied to RhoH as the presence of the inserted domain, LFSINE, at the C-terminal was shown to act as a recognition signal for lysosomal uptake and CMA-mediated degradation, while not affecting its function on both T cells and B cells [26].

Another alternative is by targeting the RhoH interacting partner, ZAP−70. RhoH mediates TCR-induced ZAP−70 activation by recruiting it to CD3ζ in the immunological synapse [23,32,34]. Additionally, RhoH was also found to promote ZAP−70-induced BCR signalling, which has been implicated in CLL [33,68,69,70]. Hence, preventing ZAP−70 recruitment to the plasma membrane may help to block excessive signalling. Several studies have identified potential ZAP−70 inhibitors that can modulate the interaction with TCR [114]. However, despite its therapeutic potential in autoimmune diseases and organ transplant rejection in vitro and in vivo, these inhibitors have never been tested in clinical trials, indicating the need for further investigation.

Alternatively, a peptide that can block or stimulate RhoH-related signalling may be used as a potential therapeutic target. The use of therapeutic peptides is an emerging targeted therapy that is often associated with rapid production and capacity for modification. However, this strategy has several drawbacks that usually arise due to their limited bioavailability and specificity [115,116]. Thus, far, no specific RhoH-therapeutic peptide is available or has been reported that may be due to the limited knowledge of RhoH functional activities.

## 5. Conclusions

Most studies identified the role of RhoH in regulating TCR signalling, but the contribution of RhoH to the BCR signalling pathway should be further elucidated. However, deregulated RhoH protein expression either through aSHM, lysosomal degradation, or transcriptional repression, has been associated with poor outcomes of B cell malignancies and immune-related diseases, such as immunodeficiency and autoimmune diseases. RhoH protein expression is significantly correlated with the aggressiveness of the malignancies and potentially involved in promoting cancer development among immune-deregulated patients. These disease associations involve the RhoH interacting partner, ZAP−70; activation regulators, Vav1 and RhoGDIs and other small GTPases, such as RhoA, Rac1 and Cdc42. RhoH also mediates cytokine release by T helper cells that may contribute to disease aetiopathogenesis.

Thus, this review suggests the important role of RhoH in both normal and disease states and presents evidence that it is an attractive target for therapeutic interventions. Nevertheless, further experimental evidence is needed to fully understand RhoH functions, especially in B cell homeostasis and other immune-related diseases.

## Figures and Tables

**Table 1:** The percentage sequence similarity shared between Rho-family GTPases.

	Rho BTB1	Rho BTB2	RhoH	Rnd1	Rnd2	Rnd3	RhoD	RhoF	RhoA	RhoC	RhoB	Wrch2	Wrch1	TC10	TCL	Cdc42	RhoG	Rac2	Rac1	Rac3
**RhoBTB1**		70	34	33	31	30	34	28	38	37	38	34	32	38	35	40	40	41	42	41
**RhoBTB2**	70		32	32	31	29	34	28	39	37	38	33	32	39	37	40	40	42	42	41
**RhoH**	34	32		29	32	36	38	33	40	40	41	41	38	40	39	42	40	40	41	40
**Rnd1**	32	32	29		53	61	37	39	41	42	41	31	32	36	34	37	37	39	39	38
**Rnd2**	31	31	32	53		63	39	41	46	47	43	28	31	36	35	37	41	40	41	39
**Rnd3**	29	29	36	61	63		37	40	48	48	47	31	32	39	35	38	41	39	42	40
**RhoD**	34	35	38	37	39	37		49	49	49	49	39	36	42	38	43	44	46	49	49
**RhoF**	28	28	33	39	41	40	49		47	48	47	36	37	46	43	43	46	50	59	47
**RhoA**	38	39	40	41	46	48	49	47		92	85	40	44	51	48	53	55	53	57	55
**RhoC**	37	37	40	42	47	48	49	48	92		85	40	44	50	49	51	55	53	57	54
**RhoB**	38	38	41	41	43	47	49	47	85	85		42	45	51	48	50	53	54	55	54
**Wrch2**	34	33	41	31	28	31	39	36	40	40	42		59	51	48	53	46	51	52	53
**Wrch1**	32	32	37	32	31	32	36	37	44	44	45	59		50	46	56	48	54	54	54
**TC10**	38	39	40	36	36	39	42	46	51	50	51	51	50		76	66	54	60	62	61
**TCL**	35	37	39	34	35	35	38	44	48	49	48	48	46	76		63	53	58	60	59
**Cdc42**	40	40	42	37	37	38	43	43	53	51	50	53	56	66	63		61	69	71	70
**RhoG**	39	40	40	37	41	41	44	46	55	55	53	46	48	54	53	60		72	72	70
**Rac2**	41	42	40	39	40	39	46	50	53	53	54	50	54	60	58	69	72		92	89
**Rac1**	42	42	41	39	41	42	49	49	57	57	55	52	54	62	60	71	72	92		93
**Rac3**	41	41	40	38	39	40	49	47	55	54	54	53	54	61	59	70	70	89	93	

**Table 2:** C-terminal sequences of the Rho-family small GTPases and their lipid modification.

Group	Rho Protein	C-Terminal Sequence	Lipid Modification	Ref
Typical	RhoA	KDGVREVFEMATRAALQARRGKKKSGCLVL	GG	[11]
	RhoB	VREVFETATRAALQKRYGSQNGCINCCKVL	GG, F, P	
	RhoC	KEGVREVFEMATRAGLQVRKNKRRRGCPIL	GG	
	Rac1	RGLKTVFDEAIRAVLCPPPVKKRKRKCLLL	GG, P	
	Rac2	RGLKTVFDEAIRAVLCPQPTRQQKRACSLL	GG	
	Rac3	RGLKTVFDEAIRAVLCPPPVKKPGKKCTVF	GG	
	RhoG	QDGVKEVFAEAVRAVLNPTPIKRGRSCILL	GG	
	Cdc42	QKGLKNVFDEAILAALEPPEPKKSRRCVLL	GG	
	TCL	AVFDEAILTIFHPKKKKKRCSEGHSCCSII	F	
	TC10	DEAIIAILTPKKHTVKKRIGSRCINCCLIT	F, P	
Atypical	RhoU	QQQPKKSKSRTPDKMKNLSKSWWKKYCCFV	P	[11]
	RhoV	EHKARLEKKLNAKGVRTLSRCRWKKFFCFV	P	
	RhoD	AVFQEAAEVALSSRGRNFWRRITQGFCVVT	F, GG	[12]
	RhoF	EDVFREAAKVALSALKKAQRQKKRRLCLLL	F, GG	
	Rnd1/ RhoS	LSKRLLHLPSRSELISSTFKKEKAKSCSIM	F	[11]
	Rnd2/ RhoN	MQRSAQLSGRPDRGNEGEIHKDRAKSCNLM	F	
	Rnd3/ RhoE	KRISHMPSRPELSAVATDLRKDKAKSCTVM	F	
	RhoH/ TTF	VFECAVRTAVNQARRRNRRRLFSINECKIF	GG, F	[11,12]
	RhoBTB1	KREREKEDIALNKHRSRRKWCFWNSSPAVA	Unknown	N/A
	RhoBTB2	KRRWLFWNSPSSPSSSAASSSSPSSSSAVV	Unknown	

Putative palmitoylated cysteines are in boldface type and coloured red and CAAX prenylation motifs are underlined. N/A: not applicable.

**Table 3:** Selected amino acid sequences of the typical and atypical small Rho GTPases.

Amino Acids 12, 59 and 61			
Group	Subfamily	Member	Sequence
Classic	Cdc42	Cdc42	**12** **59** **61** 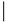 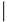 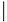
			GD**G**AV---**A**G**Q**ED
Fast-cycling	RhoU/RhoV	RhoU	GD**G**AV---**A**G**Q**ED
		RhoV	GD**G**AV---**A**G**Q**DE
	RhoD/RhoF	RhoD	GD**G**GC---**A**G**Q**DD
		RhoF	GD**G**GC---**A****G****Q**ED
GTPase defective	RhoBTB	RhoBTB−1	GD**N**AV---**F**G**D**HH
		RhoBTB−2	GD**N**AV---**F****G**DHH
	Rnd	Rnd1	GD**V**QC---**S**G**S**PY
		Rnd2	GD**A**EC---**S****G****S**SY
		Rnd3	GD**V**QC---**S**G**S**PY
	RhoH		GD**S**AV---**A**G**N**DA
**Amino acids 28**			
Classic	Rac	Rac1	**28** 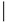
			SYTTNA**F**PGEYIP
Fast-cycling	RhoU/RhoV	RhoU	SYTTNG**Y**PTEYIP
		RhoV	SYTCNG**Y**PARYRP
	RhoD/RhoF	RhoD	VFADGA**F**PESYTP
		RhoF	VYSQGS**F**PEHYAP

Amino acid residues 12, 28, 59 and 61 are in boldface type and underlined [13].

## Data Availability

No new data were created or analyzed in this study. Data sharing is not applicable to this article.

## Abbreviations

TCR	T cell receptor
CMA	chaperone-mediated autophagy
LAT	linker of activated T cells
DLBCL	diffuse large B-cell lymphoma
aSHM	aberrant somatic hypermutation
AID	activation-induced deaminase
CLL	Chronic lymphocytic leukaemia
FL	follicular lymphoma
CSK	C-terminal Src kinase
HCL	hairy cell leukaemia
AML	acute myeloid leukaemia
PIDs	primary immunodeficiencies
SLE	systemic lupus erythematosus
BCR	B cell receptor
LCK	lymphocyte-specific protein tyrosine *kinase*
SRSF1	serine/arginine-rich splicing factor 1
GEF	guanine nucleotide exchange factors
GDI	Guanosine nucleotide dissociation inhibitor
